# Accuracy of Broad-Panel PCR-Based Bacterial Identification for Blood Cultures in a Pediatric Oncology Population

**DOI:** 10.1128/spectrum.00221-21

**Published:** 2021-07-07

**Authors:** C. D. Garner, J. Brazelton de Cardenas, S. Suganda, R. T. Hayden

**Affiliations:** a Department of Pathology, St. Jude Children’s Research Hospital, Memphis, Tennessee, USA; Memorial Sloan Kettering Cancer Center

**Keywords:** antimicrobial susceptibility testing, AST, PCR, automated, blood culture, rapid

## Abstract

Bloodstream infections are a major cause of morbidity and mortality and result in significant costs to health care systems. Rapid identification of the causative agent of bloodstream infections is critical for patient treatment and improved outcomes. Multiplex PCR systems that provide bacterial identification directly from the blood culture bottle allow for earlier detection of pathogens. The GenMark Dx ePlex blood culture identification (BCID) panels have an expanded number of targets for both identification and genotypic markers of antimicrobial resistance. The performance of the ePlex BCID Gram-negative (GN) and Gram-positive (GP) panels were evaluated in a predominantly pediatric oncology population. A total of 112 blood cultures were tested by the ePlex BCID GN and GP panels and results were compared to those from standard-of-care testing. Accuracy for on-panel organisms was 89% (CI, 76% to 95%) for the Gram-positive panel, with four misidentifications and one not detected, and 93% (CI, 82% to 98%) for the Gram-negative panel, with two misidentifications and one not detected. The results showed good overall performance of these panels for rapid, accurate detection of bloodstream pathogens in this high-risk population.

**IMPORTANCE** Bloodstream infections are a major cause of morbidity and mortality and result in significant costs to health care systems. Rapid identification of the causative agent of bloodstream infections is critical for patient treatment and improved outcomes. Multiplex PCR systems that provide bacterial identification directly from the blood culture bottle allow for earlier characterization of pathogens. The GenMark Dx ePlex blood culture identification (BCID) panels, recently cleared by the FDA, have an expanded number of targets for both identification and resistance, much larger than other, automated, broad-panel PCR assays. The performance of the ePlex BCID Gram-negative and Gram-positive panels was evaluated in a predominantly pediatric oncology population, providing a unique look at its performance in a high-risk group, where rapid diagnostic information for bloodstream infections could be of particular value for clinical care providers.

## INTRODUCTION

Bloodstream infections are a major cause of morbidity and mortality, with significant costs to society and health care systems (1, 2). Detection and identification of the causes of infection are fundamental to directing effective therapy and limiting the sequelae of these events. Typically, this has been accomplished by using liquid media blood culture systems, coupled with phenotypic identification and antimicrobial susceptibility testing (AST) (3). While these methods have a long history of use, they suffer from limits of sensitivity and prolonged time to results. The use of continuously read, automated blood culture systems over the last 3 decades has mitigated the time to detection issue and, in recent years, several systems have become widely available that can markedly reduce the time required for microbial identification once blood cultures have become positive. These include matrix-assisted laser desorption ionization–time of flight mass spectrometry (MALDI-TOF) (4), now commonly seen supplanting biochemical/phenotypic identification systems. In addition, several molecular systems provide rapid identification of infectious pathogens from positive blood culture bottles, either in targeted (single-plex) assays or in broadly multiplexed panels (5–7). These systems have in some cases markedly reduced the time between detection of a positive culture and reporting of identification results; however, until recently these panels have contained a limited number of targets—often less than a dozen each of Gram-positive and Gram-negative genospecies that can be identified.

The GenMark Dx ePlex blood culture identification (BCID) panels (GBCID), recently cleared by the FDA, have an expanded number of targets for both identification and genotypic markers of antimicrobial resistance. Bacterial identification and resistance loci in this product are split among two panels, between them encompassing 21 Gram-negative (BCID-GN panel) and 20 Gram-positive (BCID-GP panel) identifications (Table 1), with both Gram-positive antimicrobial resistance (AR) loci (*mecA*, *mecC*, *vanA*, and *vanB*) and Gram-negative AR loci (CTX-M, IMP, KPC, NDM, VIM, and OXA [OXA23 and OXA-48]). Each of the bacterial panels contains a pan-*Candida* target, which can detect four major circulating *Candida* species, while the BCID-GN and BCID-GP panels have a pan-Gram-positive and a pan-Gram-negative target (respectively), encompassing most widely known pathogenic organisms in each group. The “pan” targets were not assessed in this particular study. A third panel that is part of the ePlex BCID panel family, the ePlex fungal pathogen panel (BCID-FP), targets 15 fungal pathogens and was also not part of the present evaluation. The availability of such broad panels offers the potential for direct-from-blood culture bottle identification of a much broader variety of bacterial and fungal pathogens, along with antimicrobial resistance genes, than was previously possible. The added value of rapidly detecting important resistance loci has implications for both clinical decision making and antimicrobial stewardship. When coupled with typical, automated blood culture systems, this allows actionable identification and antimicrobial susceptibility information for a majority of positive blood cultures within 24 h of collection.

**TABLE 1:** GenMark ePlex blood culture identification (BCID) panels

Organism or resistance gene	The BCID-GN panel	The BCID-GP panel
Bacterial organisms	Acinetobacter baumannii *Bacteroides fragilis* *Citrobacter* Cronobacter sakazakii, Enterobacter cloacae complex Enterobacter (non-*cloacae* complex) Escherichia coli *Fusobacterium necrophorum* Fusobacterium nucleatum Haemophilus influenzae Klebsiella oxytoca Klebsiella pneumoniae group Morganella morganii Neisseria meningitidis *Proteus* Proteus mirabilis Pseudomonas aeruginosa Salmonella *Serratia* Serratia marcescens Stenotrophomonas maltophilia	Bacillus cereus group Bacillus subtilis group *Corynebacterium* Cutibacterium acnes (Propionibacterium acnes) *Enterococcus* Enterococcus faecalis Enterococcus faecium *Lactobacillus* *Listeria* Listeria monocytogenes *Micrococcus* Staphylococcus Staphylococcus aureus Staphylococcus epidermidis Staphylococcus lugdunensis Streptococcus Streptococcus agalactiae (GBS) Streptococcus anginosus group Streptococcus pneumoniae Streptococcus pyogenes (GAS)
Antibiotic Resistance Genes	CTX-M (*bla*CTX-M) IMP (*bla*IMP) KPC (*bla*KPC) NDM (*bla*NDM) OXA (*bla*OXA) (OXA-23 and OXA-48 groups only) VIM (*bla*_VIM_)	*mecA* *mecC* *vanA* *vanB*
Pan targets^*a*^	Pan Gram-positive Pan *Candida*	Pan Gram-negative Pan *Candida*

a Pan targets were not evaluated as part of this study.

The GBCID has only recently been FDA cleared for *in vitro* diagnostic (IVD) use. In this evaluation, the BCID-GN and BCID-GP panels used for testing were for research use only (RUO). However, it is important to note that there are no differences in the two panel constructs between the RUO versions and IVD versions that have received 510k clearance since this study was performed. Studies to-date have largely described its use in adult, general medical populations (8–11). Here, the performance of the GBCID was evaluated in a predominantly pediatric oncology population, providing a unique look at its performance in a high-risk group where rapid diagnostic information for bloodstream infections could be of particular value for clinical care providers.

## RESULTS

A total of 57 samples were tested with the BCID-GP panel; of these, 5 (1 *Kocuria* sp., 3 Staphylococcus sp., 1 *Enterococcus* sp.) yielded an invalid result (9% invalid rate). Among the 52 producing a valid result, 45 represented monomicrobial cultures and were included in the determination of accuracy (Table 2 and Table 3), and all but one were on-panel organisms. The one sample with an off-panel organism was a *Rothia* sp. (Table 3). Among monomicrobial samples, accuracy was determined to be 98% to the genus level and 89% (confidence interval [CI], 76% to 95%) to the species level when only samples that had an on-panel organism identified by standard-of-care testing (SOC) were included. Three of the five organisms not correctly identified were Streptococcus spp.; however, the BCID-GP panel was able to identify the Streptococcus genus correctly in two of the samples. Two coagulase-negative Staphylococcus spp. were identified to the genus level, but the species were not correctly identified (Table 3).

**TABLE 2:** Organisms detected correctly in monomicrobial samples

Gram-negative	No.	Gram-positive	No.
Citrobacter	1	Enterococcus faecalis	1
Escherichia coli	27	Micrococcus	2
Enterobacter cloacae complex	3	Staphylococcus aureus	3
Klebsiella pneumoniae	4	Staphylococcus epidermidis	22
Pseudomonas aeruginosa	3	Streptococcus pneumoniae	1
Serratia marcescens	1	Staphylococcus	4
Stenotrophomonas maltophilia	2	Streptococcus	6

**TABLE 3:** Discrepant samples for monomicrobial samples

Sample ID	SOC result^*a*^	ePlex BCID panel result^*a*^	WGS BLAST/WGS KMER result^*a*^^,^^*b*^
BCID-GP panel			
BCID 8	VGS	Streptococcus pneumoniae	Streptococcus mitis
BCID 30	VGS	ND	Streptococcus salivarius
BCID 35	VGS	Streptococcus pneumoniae	Streptococcus mitis
BCID 57	CoNS	Staphylococcus epidermidis	Staphylococcus hominis
BCID 69	CoNS	Staphylococcus epidermidis	Staphylococcus pasteuri
BCID 50	Rothia dentocariosa	ND	Rothia mucilaginosa ^*b*^
BCID-GN panel			
BCID 12	Escherichia coli	ND	Escherichia coli
BCID 31	Klebsiella oxytoca	Klebsiella oxytoca; Enterobacter cloacae complex	NP
BCID 43	Klebsiella pneumoniae	Klebsiella pneumoniae; Escherichia coli	NP
BCID 24	Pantoea agglomerans	ND	Pantoea vagans ^*b*^
BCID 28	Rhizobium radiobacter	ND	*Rhizobium* sp.^*b*^
BCID 70	Rahnella aquatilis	ND	Rahnella aquatilis ^*b*^
BCID 83	*Pantoea sp.*	ND	Pantoea vagans b
BCID 98	Pseudomonas putida	ND	Pseudomonas monteilii ^*b*^
BCID 104	Pseudomonas oryzihabitans	ND	Pseudomonas oryzihabitans ^*b*^

a SOC, standard of care; BCID, blood culture identification; WGS, whole-genome sequencing; VGS, viridans group streptococci; CoNS, coagulase-negative Staphylococcus spp.; ND, not detected; NP, not performed.

b Organism was not an on-panel organism.

Seven polymicrobial infections were tested with the BCID-GP panel. Four of the seven had a complete match between SOC and BCID-GP panel, two had a partial match, and one was a complete mismatch at the species level (Table 4). For both cases of partial mismatch, viridans group streptococci (VGS) was not detected. For the complete (species level) mismatch, SOC detected Staphylococcus haemolyticus and VGS and the BCID-GP panel detected Staphylococcus epidermidis and Streptococcus pneumoniae. For these cases, whole-genome sequencing (WGS) analysis confirmed the SOC identifications (Table 4). Polymicrobial cultures were not further assessed to show whether or not growth of one organism surpassed the other organism in the culture, thereby causing the bottle to flag positive and potentially resulting in a limit-of-detection challenge on the BCID-GP panel for the slower-growing organism in the bottle.

**TABLE 4:** Polymicrobial samples

Sample id	SOC result^*a*^	ePlex BCID panel result^*a*^	WGS BLAST/WGS KMER result^*a*^
BCID-GP Panel			
BCID 4	Staphylococcus haemolyticus; Candida guilliermondii	Staphylococcus	NP
BCID 19	Staphylococcus haemolyticus; VGS	Staphylococcus epidermidis; Streptococcus pneumoniae	Staphylococcus haemolyticus; Streptococcus mitis
BCID 32	Enterococcus faecium; VGS	Enterococcus faecium	NP for Enterococcus; Streptococcus mitis
BCID 68B	Staphylococcus epidermidis; Pseudomonas fluorescens	Staphylococcus epidermidis	NP
BCID 94	Staphylococcus epidermidis; VGS	Staphylococcus epidermidis; Streptococcus	NP
BCID 96A	VGS; Acinetobacter baumannii group	ND	Streptococcus mitis
CID 99	Staphylococcus epidermidis; VGS	Staphylococcus epidermidis; Streptococcus	NP
BCID-GN panel			
BCID 68A	Pseudomonas fluorescens; Staphylococcus epidermidis	ND	Pseudomonas fluorescens ^*b*^
BCID 93	Enterobacter cloacae complex; Acinetobacter baumannii complex	Enterobacter cloacae complex	Enterobacter cloacae complex not sequenced; Acinetobacter nosocomialis^*b*^
BCID 112	Stenotrophomonas maltophilia; Pseudomonas putida	Stenotrophomonas maltophilia	S. maltophilia not sequenced; Pseudomonas putida^*b*^
BCID 116	Klebsiella pneumoniae; Enterobacter cloacae complex	Klebsiella pneumoniae; Enterobacter cloacae complex	NP

a SOC, standard of care; BCID, blood culture identification; WGS, whole-genome sequencing; VGS, viridans group streptococci; CoNS, coagulase-negative Staphylococcus spp.; ND, not detected; NP, not performed.

b Organism was not an on-panel organism.

Three Staphylococcus aureus were detected in the study. The BCID-GP panel detected *mecA* for two of the samples and not the third. In all three cases, the results were confirmed correct by whole-genome sequencing. There were two *Enterococcus* spp. detected in the evaluation, one Enterococcus faecalis and one Enterococcus faecium. The BCID-GP panel did not detect *vanA* or *vanB* in either sample and these results were confirmed by whole-genome sequencing. The BCID-GP panel detected *mecA* in 21 coagulase-negative Staphylococcus spp. and WGS analysis confirmed 18 of these samples. Two samples were unavailable for confirmatory testing, and one sample did not have a *mecA* gene detected by WGS analysis.

Among the 55 samples tested with the BCID-GN panel, one (*Achromobacter* sp.) had a result of invalid (2% invalid rate). Among the 54 evaluable samples from the BCID-GN panel, 50 were monomicrobial and 44 of the 50 had on-panel results (Table 2 and Table 3). The six off-panel samples all had results of not detected with the BCID-GN panel (Table 3). Accuracy was determined to be 93% (CI, 82% to 98%) when only samples that had an on-panel organism identified by SOC were included. The BCID-GN panel did not detect one Escherichia coli, and an Escherichia coli and Enterobacter cloacae complex (detected by the BCID-GN panel) were not detected by SOC methods in two separate samples (Table 3).

Four polymicrobial samples were tested with the BCID-GN panel. One of four had a complete match. However, when off-panel organisms were excluded, the samples matched 100% for all four samples (Table 4).

Seventeen Gram-negative samples had detection of resistance markers. Of the 17, 14 had detection of CTX-M alone, two had detection of CTX-M and NDM, and one had detection of KPC and CTX-M. All results matched those of the Check-Points Assay or WGS, except the sample that had the KPC and CTX-M. For this sample, KPC and SHV-66 were the only targets detected by both the Check-Points Assay and by whole-genome sequencing.

## DISCUSSION

Results here show that the ePlex blood culture identification panels perform well in the identification of both Gram-positive and Gram-negative bacteria in positive cultures from pediatric immunocompromised patients. While numbers of individual organism types were insufficient for an assessment of accuracy by genus or species, the overall accuracy found here was 89% (CI, 76% to 95%) for Gram-positive bacteria and 93% (CI, 82% to 98%) for Gram-negative bacteria when assessing results for on-panel organisms. Identification of streptococcal and coagulase-negative staphylococcal species showed some challenges in this population; when species-level identification of this group was excluded from analysis, the overall accuracy of identification for the BCID-GP panel increased to 98%. Detection of antimicrobial resistance loci appeared to show high sensitivity and specificity, but with too few positive samples for statistical assessment.

Among monomicrobial and polymicrobial samples, there were three instances where SOC produced results of VGS and the BCID-GP panel produced results of Streptococcus pneumoniae. In all three cases, WGS analysis confirmed the identification as VGS. This difficulty in differentiating VGS from Streptococcus pneumoniae is not surprising, since they are phylogenetically similar and can be difficult to differentiate even using sequence analysis (12, 13). However, other published evaluations of this panel did not demonstrate the same inaccuracies with Streptococcus spp. (8–11). Among Staphylococcus species, there were two instances where SOC produced a result of coagulase-negative Staphylococcus species and the BCID-GP panel identified Staphylococcus epidermidis. Discrepant analysis using WGS identified Staphylococcus hominis in one sample, with the other showing Staphylococcus pasteuri. Similar issues with Staphylococcus epidermidis identification to the species level were observed in a previous study (10). The Staphylococcus misidentifications would not likely be clinically significant since they are all coagulase-negative and would have the same susceptibility testing interpretations, but they do show a potential limitation in species-level differentiation for Staphylococcus in this population.

Overall performance for identifying resistance markers was very good. In only two cases were the GBCID results not confirmed, one coagulase-negative Staphylococcus species with *mecA* and one Gram-negative species with CTX-M. In the latter case, one organism was detected by SOC, a Klebsiella pneumoniae with a KPC and a SHV mutant. In this same sample, the BCID-GN panel detected an additional organism, Escherichia coli, and an additional resistance marker, CTX-M. Potentially, a CTX-M-positive Escherichia coli could have been present in the blood culture bottle but not identified from the subculture.

The study was limited by low numbers, but these were felt sufficient for proof-of-concept of the ePlex BCID panels in this so-far poorly studied and high-risk patient population. Further work is needed to assess the cause of discrepant results seen in staphylococcal and streptococcal identification; these have not been widely reported and may be population-specific. They do not obviate the utility of the assay, which we have chosen to implement with only genus-level streptococcal identifications and without differentiation of the staphylococcal species in question. Results of resistance loci analysis appear promising, but definitive evaluation must also await larger sample sets.

The ePlex BCID panels appear to have a high overall accuracy in this predominantly immunocompromised pediatric population. The breadth of bacterial organisms covered will permit rapid identification in over 90% of positive blood cultures, based on past distributions of infectious agents. This, together with rapid detection of high-interest AR loci creates rapid access to actionable information for clinical care providers, allowing informed decisions regarding care and best use of therapeutics, contributing to ongoing antimicrobial stewardship efforts. While further work is needed to assess the impact on care, antimicrobial use, and outcome, findings here support the accuracy of this test in high-risk, high-acuity pediatric patients.

## MATERIALS AND METHODS

### Study design.

A prospective evaluation was performed between September 2018 and March 2019 to evaluate the GenMark Dx ePlex RUO blood culture identification (BCID) Gram-positive and Gram-negative panels. A total of 112 blood cultures collected using Bactec liquid media bottles (Bactec Peds Plus/F Medium, Bactec Plus Aerobic/F Medium, Bactec Plus Anaerobic/F Medium, Becton-Dickenson, Franklin Lakes, New Jersey) were included. Samples flagging positive on the Bactec FX (Becton-Dickenson) were processed per standard of care (SOC) with Gram stain and subculture. After determining the Gram stain results, a 1-ml aliquot of blood was removed for further testing with the GBCID. Samples that were tested with the GBCID within the previous 7 days were excluded from this evaluation, because they were classified as occurring within the same episode. Samples that were Gram-positive were tested using the BCID-GP panel, samples that were Gram-negative were tested using the BCID-GN panel, and samples that were Gram-variable were tested using both panels. Results from the GBCID for identification were compared to SOC methodologies, which primarily included Vitek MS and Vitek 2 Compact (bioMérieux, Marcy l'Etoile, France). Discrepant results were resolved using whole-genome sequencing (WGS) analyzed using BLAST or KMER analysis. Results from the GBCID for antimicrobial resistance markers *mecA*, *mecC*, *vanA*, and *vanB* were compared to WGS results. For Staphylococcus aureus and *Enterococcus* sp., all samples were sequenced to look for the presence or absence of *mecA*, *mecC*, *vanA*, and *vanB*. For coagulase-negative Staphylococcus sp., WGS was performed only for samples where *mecA* was detected, for confirmatory purposes. Check-Points MicroArray results and WGS were used for confirmation of CTX-M, KPC, NDM, OXA (OXA-23 and OXA-48), and VIM in Gram-negative samples.

### Standard-of-care methods.

Positive blood cultures were subcultured on solid medium for 16 to 24 h prior to identification on the Vitek MS or Vitek 2 Compact. For Vitek MS, a 1-μl inoculation loop was used to add cellular material to the individual spot of the Fleximass-DS slide, then Matrix DHB was added to the spot. Spectral data were collected and analyzed on the Vitek analysis software, Myla (bioMérieux). For the Vitek 2 Compact, isolated colonies from the subculture were used to make a saline McFarland solution of 0.5 to 0.63. Appropriate test cards (Vitek 2 GP or Vitek 2 GN) were run as per the Vitek 2 operator's manual, with Vitek Observa software (bioMérieux).

### GenMark Dx ePlex blood culture identification panels.

Blood culture aliquots (1 ml) were stored at room temperature until testing using the GBCID panels. All samples were tested within 7 days of positivity on the Bactec FX. An aliquot (50 μl) of positive blood culture was placed in the loading port of the GCBID cartridge. The sample loading port was immediately closed. The sample was scanned by the barcode reader and then loaded into an available bay on the ePlex instrument for testing.

### Nucleic acid extraction.

**(i) Gram-negative isolates.** Extraction was done by lysing several colonies from purity plates in 2 ml of NucliSENS easyMAG lysis buffer (bioMérieux). This was incubated for 10 min, then extracted using the bioMérieux nucliSENS easyMAG nucleic acid extractor (bioMérieux), Specific A protocol. The final DNA elution was 100 μl in NucliSENS easyMAG elution buffer (bioMérieux) and was stored at −20°C until use.

**(ii) Gram-positive isolates.** Colonies were suspended to make a 1.5 McFarland in tryptic soy broth (TSB). An aliquot (600 μl) of the suspension was added to 2 ml of Lysing Matrix B with 0.1 mm silica spheres, split between 2 tubes (MP Biomedicals). The lysis tubes were vortexed at 6 m/second for 30 s using the FastPrep 24 5G (MP Biomedicals). The vortexing step was repeated with 5-min incubations on ice after each vortexing. samples were centrifuged, and 400 μl of the resulting supernatant was mixed with 10 μl proteinase K (600 mAU/ml solution) and incubated at 55°C for 15 min. Final purification was done using the EZ1 DNA tissue kit (Qiagen) for the EZ1 Advanced XL instrument and EZ1 Advanced XL DNA tissue card (Qiagen) with a 50-μl elution volume, and DNA was stored at −20°C until use.

### Check-Points MicroArray assay.

To detect and identify extended-spectrum beta-lactamases and carbapenemases, the Check-MDR CT103XL assay (Check-Points BV, Wageningen, Netherlands) was performed per manufacturer’s instructions and as previously described by others (14–17). Briefly, DNA extract was ligated to beta-lactamase-specific probes, amplified in a multiplex PCR, hybridized to specialized microarray tubes, and visualized and analyzed using the Check-Points Tube Reader and software (Check-Points BV). Isolates were identified as carrying variants to extended-spectrum β-lactamase (ESBL), AmpC, and carbapenemase genes.

### Isolate sequencing.

An individual sequencing library was prepared for each isolate using the NexteraXT DNA sample preparation kit (Illumina, San Diego, CA, USA). Then, isolates were pooled and multiplexed for paired-end sequencing on a single MiSeq instrument, using the MiSeq reagent kit v3 150 bp (Illumina). Libraries were assembled by SeqSphere+ software version 4.1.9 (Ridom GmbH, Muenster, Germany) for automated quality trimming and assembly. The resulting FASTA files were uploaded to the KmerFinder 3.1 on the Center for Genomic Epidemiology and the top Kmer result for each isolate was recorded (18–20). Each isolate was also analyzed by the Comprehensive Antibiotic Resistance Database (CARD) and the presence of *mecA*, *mecC*, and *vanA* was determined (17, 21–23).

### Data analysis.

This quality improvement study was reviewed by the institutional Office of Human Subjects Protection and it was determined it did not meet the definition of research, thus, informed consent was not required. The percent invalid rate was determined for each panel by taking the proportion of invalid tests (those failing to produce a result)/total number tested. Accuracy was calculated as the proportion of organisms identified correctly when samples with SOC results, not on the panel, were excluded. Accuracy was determined for monomicrobial infections only; Polymicrobial infections (samples with two organisms or more) were not included in this analysis, but the results were described. Analysis of the pan-target results was considered beyond the scope of this evaluation, as it was determined prior to analyzing the results of the evaluation that the pan target results would not be used at our institution for patient care purposes.

## References

[1] Arefian H, Heublein S, Scherag A, Brunkhorst FM, Younis MZ, Moerer O, Fischer D, Hartmann M. 2017. Hospital-related cost of sepsis: a systematic review. J Infect 74:107–117. doi:10.1016/j.jinf.2016.11.006. 27884733

[2] Hajj J, Blaine N, Salavaci J, Jacoby D. 2018. The “centrality of sepsis”: a review on incidence, mortality, and cost of care. Healthcare (Basel) 6:90. doi:10.3390/healthcare6030090. 30061497 PMC6164723

[3] Opota O, Croxatto A, Prod'hom G, Greub G. 2015. Blood culture-based diagnosis of bacteraemia: state of the art. Clin Microbiol Infect 21:313–322. doi:10.1016/j.cmi.2015.01.003. 25753137

[4] Croxatto A, Prod'hom G, Greub G. 2012. Applications of MALDI-TOF mass spectrometry in clinical diagnostic microbiology. FEMS Microbiol Rev 36:380–407. doi:10.1111/j.1574-6976.2011.00298.x. 22092265

[5] Dodemont M, De Mendonca R, Nonhoff C, Roisin S, Denis O. 2014. Performance of the Verigene Gram-negative blood culture assay for rapid detection of bacteria and resistance determinants. J Clin Microbiol 52:3085–3087. doi:10.1128/JCM.01099-14. 24899026 PMC4136123

[6] Huang HS, Tsai CL, Chang J, Hsu TC, Lin S, Lee CC. 2018. Multiplex PCR system for the rapid diagnosis of respiratory virus infection: systematic review and meta-analysis. Clin Microbiol Infect 24:1055–1063. doi:10.1016/j.cmi.2017.11.018. 29208560 PMC7128951

[7] Salimnia H, Fairfax MR, Lephart PR, Schreckenberger P, DesJarlais SM, Johnson JK, Robinson G, Carroll KC, Greer A, Morgan M, Chan R, Loeffelholz M, Valencia-Shelton F, Jenkins S, Schuetz AN, Daly JA, Barney T, Hemmert A, Kanack KJ. 2016. Evaluation of the FilmArray blood culture identification panel: results of a multicenter controlled trial. J Clin Microbiol 54:687–698. doi:10.1128/JCM.01679-15. 26739158 PMC4767991

[8] Carroll KC, Reid JL, Thornberg A, Whitfield NN, Trainor D, Lewis S, Wakefield T, Davis TE, Church KG, Samuel L, Mills R, Jim P, Young S, Nolte FS. 2020. Clinical performance of the novel GenMark Dx ePlex blood culture ID Gram-positive panel. J Clin Microbiol 58:e01730-19. doi:10.1128/JCM.01730-19. 31996444 PMC7098771

[9] Huang TD, Melnik E, Bogaerts P, Evrard S, Glupczynski Y. 2019. Evaluation of the ePlex blood culture identification panels for detection of pathogens in bloodstream infections. J Clin Microbiol 57:e01597-18. doi:10.1128/JCM.01597-18. 30487304 PMC6355516

[10] Oberhettinger P, Zieger J, Autenrieth I, Marschal M, Peter S. 2020. Evaluation of two rapid molecular test systems to establish an algorithm for fast identification of bacterial pathogens from positive blood cultures. Eur J Clin Microbiol Infect Dis 39:1147–1157. doi:10.1007/s10096-020-03828-5. 32020397 PMC7225181

[11] Bryant S, Almahmoud I, Pierre I, Bardet J, Touati S, Maubon D, Cornet M, Richarme C, Maurin M, Pavese P, Caspar Y. 2020. Evaluation of microbiological performance and the potential clinical impact of the ePlex((R) blood culture identification panels for the rapid diagnosis of bacteremia and fungemia. Front Cell Infect Microbiol 10:594951. doi:10.3389/fcimb.2020.594951. 33324578 PMC7726344

[12] Kawamura Y, Hou XG, Sultana F, Miura H, Ezaki T. 1995. Determination of 16S rRNA sequences of Streptococcus mitis and Streptococcus gordonii and phylogenetic relationships among members of the genus Streptococcus. Int J Syst Bacteriol 45:406–408. doi:10.1099/00207713-45-2-406. 7537076

[13] Park HK, Yoon JW, Shin JW, Kim JY, Kim W. 2010. rpoA is a useful gene for identification and classification of Streptococcus pneumoniae from the closely related viridans group streptococci. FEMS Microbiol Lett 305:58–64. doi:10.1111/j.1574-6968.2010.01913.x. 20158524

[14] Bogaerts P, Cuzon G, Evrard S, Hoebeke M, Naas T, Glupczynski Y. 2016. Evaluation of a DNA microarray for rapid detection of the most prevalent extended-spectrum beta-lactamases, plasmid-mediated cephalosporinases and carbapenemases in Enterobacteriaceae, Pseudomonas and Acinetobacter. Int J Antimicrob Agents 48:189–193. doi:10.1016/j.ijantimicag.2016.05.006. 27374747

[15] Cunningham SA, Vasoo S, Patel R. 2016. Evaluation of the Check-Points Check MDR CT103 and CT103 XL microarray kits by use of preparatory rapid cell lysis. J Clin Microbiol 54:1368–1371. doi:10.1128/JCM.03302-15. 26888905 PMC4844729

[16] Cuzon G, Naas T, Bogaerts P, Glupczynski Y, Nordmann P. 2012. Evaluation of a DNA microarray for the rapid detection of extended-spectrum beta-lactamases (TEM, SHV and CTX-M), plasmid-mediated cephalosporinases (CMY-2-like, DHA, FOX, ACC-1, ACT/MIR and CMY-1-like/MOX) and carbapenemases (KPC, OXA-48, VIM, IMP and NDM). J Antimicrob Chemother 67:1865–1869. doi:10.1093/jac/dks156. 22604450

[17] Guitor AK, Raphenya AR, Klunk J, Kuch M, Alcock B, Surette MG, McArthur AG, Poinar HN, Wright GD. 2019. Capturing the resistome: a targeted capture method to reveal antibiotic resistance determinants in metagenomes. Antimicrob Agents Chemother 64:e01324-19. doi:10.1128/AAC.01324-19. 31611361 PMC7187591

[18] Clausen P, Aarestrup FM, Lund O. 2018. Rapid and precise alignment of raw reads against redundant databases with KMA. BMC Bioinformatics 19:307. doi:10.1186/s12859-018-2336-6. 30157759 PMC6116485

[19] Hasman H, Saputra D, Sicheritz-Ponten T, Lund O, Svendsen CA, Frimodt-Moller N, Aarestrup FM. 2014. Rapid whole-genome sequencing for detection and characterization of microorganisms directly from clinical samples. J Clin Microbiol 52:139–146. doi:10.1128/JCM.02452-13. 24172157 PMC3911411

[20] Larsen MV, Cosentino S, Lukjancenko O, Saputra D, Rasmussen S, Hasman H, Sicheritz-Ponten T, Aarestrup FM, Ussery DW, Lund O. 2014. Benchmarking of methods for genomic taxonomy. J Clin Microbiol 52:1529–1539. doi:10.1128/JCM.02981-13. 24574292 PMC3993634

[21] Alcock BP, Raphenya AR, Lau TTY, Tsang KK, Bouchard M, Edalatmand A, Huynh W, Nguyen AV, Cheng AA, Liu S, Min SY, Miroshnichenko A, Tran HK, Werfalli RE, Nasir JA, Oloni M, Speicher DJ, Florescu A, Singh B, Faltyn M, Hernandez-Koutoucheva A, Sharma AN, Bordeleau E, Pawlowski AC, Zubyk HL, Dooley D, Griffiths E, Maguire F, Winsor GL, Beiko RG, Brinkman FSL, Hsiao WWL, Domselaar GV, McArthur AG. 2019. CARD 2020: antibiotic resistome surveillance with the comprehensive antibiotic resistance database. Nucleic Acids Res 48:D517–D525. doi:10.1093/nar/gkz935. PMC7145624 31665441

[22] Jia B, Raphenya AR, Alcock B, Waglechner N, Guo P, Tsang KK, Lago BA, Dave BM, Pereira S, Sharma AN, Doshi S, Courtot M, Lo R, Williams LE, Frye JG, Elsayegh T, Sardar D, Westman EL, Pawlowski AC, Johnson TA, Brinkman FS, Wright GD, McArthur AG. 2017. CARD 2017: expansion and model-centric curation of the comprehensive antibiotic resistance database. Nucleic Acids Res 45:D566–D573. doi:10.1093/nar/gkw1004. 27789705 PMC5210516

[23] McArthur AG, Waglechner N, Nizam F, Yan A, Azad MA, Baylay AJ, Bhullar K, Canova MJ, De Pascale G, Ejim L, Kalan L, King AM, Koteva K, Morar M, Mulvey MR, O'Brien JS, Pawlowski AC, Piddock LJ, Spanogiannopoulos P, Sutherland AD, Tang I, Taylor PL, Thaker M, Wang W, Yan M, Yu T, Wright GD. 2013. The comprehensive antibiotic resistance database. Antimicrob Agents Chemother 57:3348–3357. doi:10.1128/AAC.00419-13. 23650175 PMC3697360

